# The Impact of a Debriefing Process Group During the Psychiatry Clerkship

**DOI:** 10.1007/s40596-025-02200-z

**Published:** 2025-08-27

**Authors:** Andrew White, Elizabeth Britt DeVore, Elissa H. Patterson, Scott Mariouw

**Affiliations:** University of Michigan Medical School, Ann Arbor, MI USA

To the Editor:

During the core psychiatry clerkship, medical students are confronted with emotionally charged clinical scenarios including suicide attempts and severe patient agitation. These exposures have been positively associated with endpoint levels of depression and other stress symptoms [1]. Lack of insight into and avoidance of clinical experiences to which students assign a negative emotional valence have also been found to impair professional development [2]. Strategies to process these experiences are needed to minimize their negative impact on student wellbeing and encourage their effective integration into developing professional identities. There is emerging evidence for the benefit of Balint groups in medical student education, including a recent Strengths, Weaknesses, Opportunities, and Threats (SWOT) analysis of 2–3 virtual sessions [3]. As a step toward building support for medical students, our group took the initiative to trial a single-session debriefing process group for second-year medical students rotating through the core psychiatry clerkship at the University of Michigan during the 2023–2024 clerkship cycle. To assess usefulness and inform further development of the program, students were asked to complete an anonymous post-group survey about the experience.

All medical students assigned to complete the core psychiatry clerkship during the 2023–2024 academic year at the University of Michigan were eligible. Attendance was mandatory, though the participants were reminded that personal disclosure was voluntary. Group leaders emphasized confidentiality of topics discussed in the session unless the student wished to escalate the concern, or the concern clearly demonstrated imminent risk to the student or others involved. The debriefing process group was an hour long in-person educational session structured as 15 min of didactics on transference and countertransference followed by facilitated discussion of individual experiences. The group facilitators involved in the sessions did not provide clinical supervision or contribute to the grading of students to eliminate potential conflicts of interest. Data on student reactions to the group was collected using a Qualtrics questionnaire (Qualtrics, Provo, UT) comprising 11 total questions (9 quantitative items; 2 qualitative items) about previous clinical exposure to emotionally challenging events, perceived impact on personal wellness, the educational value of the process group, student comfort participating in the group, and suggestions for improvement.

We are pleased to share that 11 process groups were facilitated during the 2023–2024 clerkship cycle, and of 155 students who rotated through the clerkship, 116 (75%) completed the questionnaire. The need for the group was readily confirmed by the fact that 86% of respondents reported experiencing an emotionally challenging scenario in the clinical setting. Of note, 23% agreed that these exposures negatively impacted their personal wellness. After engaging in the debriefing process group, 62% agreed that they felt better equipped to process difficult clinical situations, and 83% agreed that the session was valuable for their training and professional development. Eighty-seven percent agreed that this debriefing group should be part of the psychiatry clerkship and 84% agreed that a similar session should be held in other clerkships. This provides an avenue for psychiatry training programs to take initiative to improve medical student wellbeing broadly, even beyond the psychiatry clerkship.

The participants’ open responses reflected that many valued having a psychologically safe space, the ability to be vulnerable around peers, and the opportunity to share experiences. One of the participants shared “I felt alone in my experiences and like maybe I was the only one feeling unprepared in interviews etc. and this helped me feel supported and like we’re all going through the same thing.” While a majority of students benefitted from engaging with the process group, improving the impact to more participating students highlights an area for growth in this programming. Student suggestions for improvement included offering the session earlier in the clerkship, having even smaller break-out groups rather than a larger 12–15 student group, and having more time or additional sessions within the clerkship curriculum.

Our pilot program is the first, to our knowledge, that incorporates a stand-alone in-person intervention of this kind during the psychiatry clerkship. This differs from previous reports on Balint groups as this is a single session, highlighting that a lower resource, one-time intervention can have a meaningful impact. Students viewed the debriefing process group as valuable with respect to their professional development, which is important as student engagement is core to the functioning and benefit of this intervention. Encouraging and protecting time for students to have a safe space to share difficult experiences with peers early in their medical training will hopefully model and support the continuation of this practice as students transition into even more demanding clinical settings in residency.

We share information about our pilot intervention here, to encourage other medical schools to consider experimenting with the inclusion of a debriefing process group as an integral part of the psychiatry clerkship curriculum. Students indicated that similar debriefing sessions may be beneficial in other clerkships as well. We see this work as a starting point for larger-scale implementation and research on debriefing process groups throughout the entire clerkship year to improve overall medical student education and professional development.

## Data Availability

The dataset generated and analyzed in the study is not publicly available due to privacy concerns. The data is available from the corresponding author upon reasonable request.
